# Planning for scale: analysis of adaptations and contextual factors influencing scale-up of the QUALI-DEC intervention to optimize caesarean section use

**DOI:** 10.1186/s43058-025-00737-6

**Published:** 2025-05-21

**Authors:** Soha El-Halabi, Claudia Hanson, Alexandre Dumont, Amanda Cleeve, Helle Mölsted Alvesson, Charles Kaboré, Guillermo Carroli, Pisake Lumbiganon, Quoc Nhu Hung Mac, Ana Pilar Betran, Kristi Sidney Annerstedt, Meghan A. Bohren, Karen Zamboni

**Affiliations:** 1 Department of Global Public Health, Karolinska Institutet, Stockholm, Sweden; 2 Department of Disease Control, London School of Hygiene & Tropical Medicine, London, England; 3 Centre of Excellence for Women and Child Health, Aga Khan University, Nairobi, Kenya; 4 Université Paris Cité, IRD, Inserm, Ceped, 75006 Paris, France; 5 Department of Women’s and Children’s Health, Karolinska Institutet, and Karolinska University Hospital, Stockholm, Sweden; 6 Institut de Recherche en Sciences de La Santé (IRSS), Ouagadougou, Burkina Faso; 7 Centro Rosarino de Estudios Perinatales, Rosario, Argentina; 8 Department of Obstetrics and Gynaecology, Faculty of Medicine, Khon Kaen University, Khon Kaen, Thailand; 9 Pham Ngoc Thach University, Ho Chi Minh City, Vietnam; 10 Department of Sexual and Reproductive Health and Research, UNDP/UNFPA/UNICEF/WHO/World Bank Special Program of Research, Development and Research Training in Human Reproduction (HRP), World Health Organization, Geneva, Switzerland; 11 Gender and Women’s Health, Nossal Institute for Global Health, School of Population and Global Health, University of Melbourne, Melbourne, Australia

**Keywords:** Scalability, Scale-up, Implementation research, Intervention, Assessment, Adaptations, Formative research, Maternal health, Caesarean section

## Abstract

**Background:**

Researchers are encouraged to plan for scale through purposeful and guided assessment of scalability of an intervention. This study analysed factors potentially influencing scale-up and synthesised early adaptations of the QUALI-DEC intervention aiming to improve the appropriate use of caesarean section. The intervention consists of opinion leader engagement, audit and feedback for caesarean section, a tool to help women make an informed decision on the mode of birth, and labour companionship.

**Methods:**

We conducted a framework analysis, which was guided by the scalability assessment framework by Zamboni et al., a 34-item checklist with a three-point scale. We used data from the formative research including a document review, hospital readiness assessment and qualitative interviews conducted between March 2019 and May 2020 in 32 facilities across Argentina, Burkina Faso, Thailand, and Viet Nam. Data were deductively coded based on the four dimensions of the scalability framework. Our findings were validated with implementing partners across countries.

**Results:**

We identified the perceived relevance of the intervention by women and providers and the presence of relevant key clinical guidelines as factors that may ease scalability of QUALI-DEC. Labour companionship and the decision-analysis tool were perceived as harder to scale-up and requiring additional changes to existing healthcare structures. Most of the study facilities reported high workload and time constraints as implementation barriers. Thailand was the only country with a national policy to reduce unnecessary caesarean sections. Legal disputes were common and followed a structured process in Thailand and Argentina, which may support preference of caesarean section due to fear of litigation. Early adaptations included development, revision and translation of educational material, monetary compensation of opinion leaders and reaching consensus on clinical guidelines to be used across hospitals, most of which are deemed conducive to scale up.

**Conclusions:**

Planning for scale-up is a key feature of the QUALI-DEC intervention. Scale-up may not be guaranteed at this point of the intervention since effectiveness and cost-effectiveness are not demonstrated yet. However, the investment in studying scale-up opportunities is a core contribution to implementation research. This exercise informed implementation and scale-up strategies of the QUALI-DEC intervention.

**Supplementary Information:**

The online version contains supplementary material available at 10.1186/s43058-025-00737-6.

Contributions to the literature

1. Researchers are encouraged to design for scale instead of expecting random diffusion of interventions. Thus, assessing the scalability of interventions is becoming a more important exercise.
2. We identified positive factors to ease the scalability of the QUALI-DEC intervention including its relevance and presence of clinical protocols. Barriers such as fear of litigation and lack of physical space were perceived as hindering factors towards its implementation.
3. These findings informed future implementations including adaptations to make the intervention more fit to context and scale-up strategies within the QUALI-DEC intervention

## Background

Evidence suggests that demonstrating intervention effectiveness on its own will not drive scale-up [1, 2]. In response, researchers are encouraged to move away from the spontaneous diffusion of a successful intervention, to rather embedding a guided and deliberate effort to take the intervention to scale during implementation [3]. Planning for scale is important to amplify the impact of evidence-based interventions [3, 4] and factors that facilitate scale have been synthesised in several frameworks, toolkits and evidence scans [1, 4–8]. We illustrate an approach to planning for scalability, prior to implementation, using the QUALI-DEC intervention study as an example.

### The QUALI-DEC intervention study

The QUALIty DECision-making by women and providers for appropriate use of CS (QUALI-DEC) intervention study aims to design, adapt and test a multifaceted intervention to address the rising rates of caesarean section [9]. A consortium of researchers initiated the QUALI-DEC intervention study combining four key interventions: (1) opinion leaders (influential obstetricians in each hospital) to implement evidence-based clinical guidelines; (2) audit and feedback using information from the Robson classification, (3) a decision-analysis tool to help women make informed decisions on mode of birth; and (4) implementation of WHO recommendations on labour companionship. The audit and feedback component builds on the Robson Ten Group Classification System [10], to analyse caesarean section rates in hospitals and evidence-based clinical algorithms on labour management, which were adapted and adopted by implementing partners in the participating countries. Implementing partners, who in this paper refer to the implementing teams at a country level, consist of principal investigators, opinion leaders, healthcare providers, social scientists, and communication officers.

The intervention is implemented in 32 hospitals in Argentina, Burkina Faso, Thailand, and Viet Nam. The start of implementation was marked by the completion of a stakeholder training workshop for the opinion leaders and data collectors in all participating countries. We considered this to be our point of departure as the training aimed to introduce the QUALI-DEC intervention and further refine it with implementing partners through a participatory approach.

A key component of the QUALI-DEC intervention is knowledge translation (KT) to promote intervention scale-up. KT plans were developed at a country level prior to the start of implementation: implementers conducted a stakeholder analysis in each country, segmenting target groups based on their interests, attitudes, and desired role in the intervention uptake and scale-up, to identify key advocacy priorities for each type of stakeholder. The KT plans included six sections: (1) vision of what needs to be changed based on the contextual characteristics, (2) general objectives of the KT process, (3) evidence to be transferred, (4) key actors, based on a stakeholder mapping exercise, (5) relevant KT strategies and activities for each stakeholder group identified, and (6) key actors to be involved in the KT preparation. The KT plans were implemented throughout the duration of the QUALI-DEC intervention, led, and revised regularly by a dedicated team member in each country and managed by the scientific coordinator.

This study is part of the QUALI-DEC evaluation composed of the effectiveness, cost-effectiveness and a nested theory-based process evaluation aiming to assess for whom and how the QUALI-DEC intervention worked, which is described in detail elsewhere [9, 11]. A key component of the process evaluation [11] is the aspect of scalability i.e. designing for scale, which is the focus of this paper. We aimed to analyse contextual factors that may impact scale-up opportunities of the QUALI-DEC intervention, before the start of implementation. Additionally, we documented early adaptations to the QUALI-DEC intervention that were presumed to ease scalability.

## Methods

This study was a cross-country analysis, mapping contextual factors influencing scalability of the QUALI-DEC intervention in Argentina, Burkina Faso, Thailand and Viet Nam. The countries and partners were selected harnessing previous research collaborations and awareness of CS rates with willingness to take action to reduce them [9]. We defined scalability as a health intervention’s ability to be expanded to a bigger population under real world conditions and retain its effectiveness [6]. We followed the standards for reporting qualitative research, when applicable, to report on the different sections of our study (see Additional file 1).

### Study setting

Included hospitals consisted of a mix of 32 tertiary, secondary and primary health facilities (Table 1). Criteria of selection are detailed elsewhere [9]. Only two out of the 32 facilities were private, both in Viet Nam, and 22 were teaching hospitals, across all countries. Average annual births ranged from approximately 1500 births in study facilities in Argentina to more than 10,000 annual births in Viet Nam. The country level percentage of births by caesarean sections was lowest in study facilities in Burkina Faso (5.3%) and highest in Thailand (32.7%) [9].

**Table 1:** Characteristics of study countries and facilities

	**Argentina**	**Burkina Faso**	**Thailand**	**Viet Nam**
**Country level indicators**				
Country income group	Upper-middle income	Low income	Upper-middle income	Middle income
Maternal mortality ratio^1^ (2020)	45 (38–53)	264 (169–394)	29 (24–34)	124 (81–190)
% of births by caesarean Sect. ^2^	29.1 (2011)	5.3 (2014)	32.7 (2016)	27.5 (2014)
**Facility level indicators**				
Facility type				
* Public*	8	8	8	6
* Private*	0	0	0	2
Referral level				
* Primary*	0	2	0	0
* Secondary*	3	4	1	7
* Tertiary*	5	2	7	1
Teaching facility	8	2	8	4
Average annual births^3^ (2020)	1528	3893	4045	10,641
Type of consent for CS	Written	Verbal	Written	Written
Medical records				
* Electronic*	8	0	4	1
* Paper-based*	0	8	4	7

^1^Expressed per 100,000 live births [12]

^2^Reflecting latest estimates on a national level from WHO data portal [13]

^3^Averages of 8 facilities based on facility readiness assessment

### Data sources

We used data from the QUALI-DEC formative research that was conducted between March 2019 and May 2020 in all four study countries [14]. The formative research included three components: (i) a document review, to map local policies and practices in the four countries; (ii) a readiness assessment, to assess contextual factors of participating facilities and (iii) qualitative interviews with women, healthcare providers and managers, to understand the relevance and feasibility of implementation and the legal context [12–19].

We used document reviews to understand the organization of health systems, including the financing and legal context, as well as available guidelines and protocols. These were complemented by facility readiness assessments to provide insight on physical layout, protocols, and guidelines for clinical care during labour and childbirth, audit and feedback and labour companionship practices in the 32 study facilities. Summaries of qualitative interviews with opinion leaders, healthcare providers and women were analysed to understand their perception of the intervention’s relevance, the role of opinion leaders and the national legal context [12, 13, 15]. Summaries of interviews were developed by implementing partners and translated to English language.

**Fig. 1:**
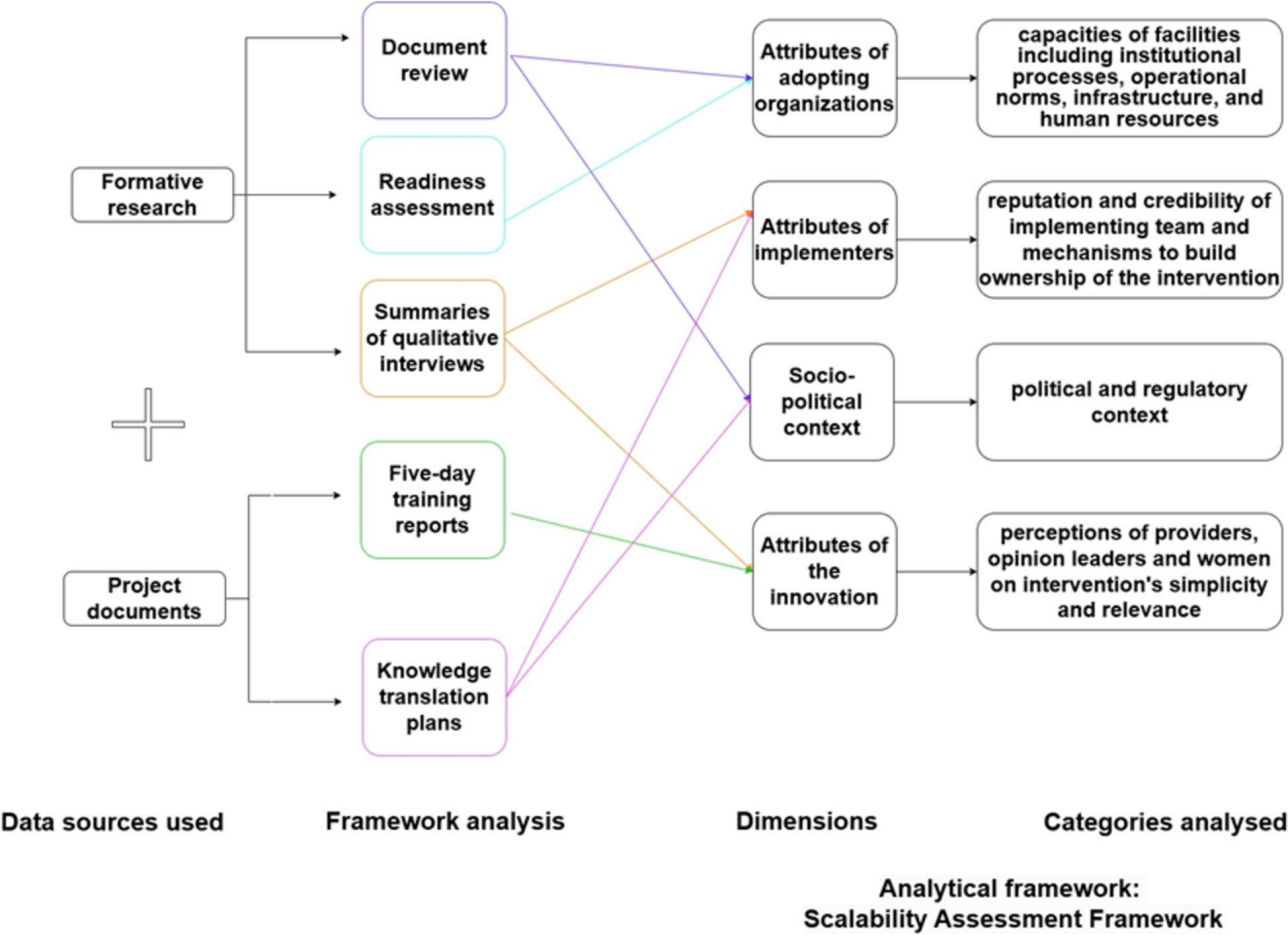
Data sources used to analyse the dimensions of the analytical framework

Additionally, we used the reports of the stakeholder’s training workshop held in each country between August 2021 (Burkina Faso) and July 2022 (Argentina) to integrate information on the feasibility of implementation, and to map adaptations agreed upon during the workshops. We also reflected on the first version of each country KT plan, developed prior to the stakeholder’s training, to understand the range of collaborations and stakeholders involved, using data on stakeholder mapping and relevant strategies to engage each stakeholder group. Figure 1 illustrates the data sources used to populate the various dimensions of the scalability assessment framework. The type of information extracted from each source is provided as an additional file (see additional file 2).

### Analysis

Our analysis was guided by the scalability assessment framework developed by Zamboni et al. [3]. The framework was developed through a rapid review, synthesis and adaptations of available scale-up frameworks [20] and was incorporated in QUALI-DEC’s study protocol [9]. It summarises factors that are relevant to scaling-up interventions based on identified scalability tools. Factors are grouped into four dimensions including (i) attributes of innovation; (ii) attributes of implementers; (iii) attributes of adopting organizations and, (iv) the socio-political context.

The scalability assessment framework comprises a checklist with 34 items that are scored on a three-point scale (easier, neutral or harder). The purpose of the three-point scale is to reflect on challenges and opportunities for scale-up rather than answer the question of whether scale-up is possible or not. We were able to assess 25 out of 34 scalability items, more details on the analysed categories and items are provided as Additional file 3 (see Additional file 3).

We conducted a framework analysis following the Framework Method approach. We organized and interpreted the data using the scalability assessment as our analytical framework [21]. We deductively coded data based on the categories of the scalability assessment framework. We used the framework’s existing definitions without any modifications. The analysis of interviews with women and providers were restricted to manifest content (i.e. summaries of what participants had said in relation to particular interview topics). Coding was performed by the first author and cross-checked with the last author. We included data on the perspectives of the intervention’s relevance of each intervention component and legal context. Data related to contextual factors were interpreted according to their ability to influence the scale-up of the QUALI-DEC intervention i.e. making the QUALI-DEC scale-up easier, neutral or harder.

We documented adaptations at a country level. We referred to these adaptations as *early adaptations*, given that they were introduced before the formal start of implementation (i.e. during the stakeholder training provided as part of the QUALI-DEC intervention). We considered adaptations to be any intentional refinement or enhancement to the content or the delivery of the intervention, without changing the essence of the intervention, introduced to better fit the local context. We used training session reports to identify early adaptations. Adaptations were identified and presented per the intervention’s four components.

The perspectives of the entire research consortium were used to dispute preliminary findings and achieve consensus. We reviewed and discussed our findings during an in-person workshop session in September 2022. The workshop session, led by the first and last authors, included a presentation of preliminary findings followed by feedback and input from various consultations: 1) a consultation with the entire consortium of researchers, and 2) representatives from each country team independently. Additionally, partners and researchers discussed and brainstormed on various potential scale-up scenarios of the QUALI-DEC intervention at a country level.

## Results

### Pre-implementation assessment of feasibility of scale-up

The main factors that may influence scale-up under each attribute are summarized in Table 2 and further elaborated thereafter.

**Table 2:** Main factors that may influence scale-up

Dimension	Factors that may influence scale-up	Influence on scale-up (easier, neutral, harder)
**Attributes of intervention**	• QUALI-DEC is perceived to respond to an important problem (high rates of CS) according to healthcare providers, women and companions	Easier
	• Some QUALI-DEC components (i.e. audit and feedback for CS) are add-ons to an already existing practice or have no competing solutions (i.e. DAT)	Easier
	• Each QUALI-DEC intervention component is proven effective, but we do not know the effectiveness of the combined components yet or ways to optimize implementation	Neutral
**Attributes of implementers**	• Opinion leaders and country Principal Investigators are trusted and have good reputations within their settings	Easier
	• Financial incentives for opinion leaders under the QUALI-DEC study may be not sustainable beyond the scope of the study	Harder
	• Local stakeholders not involved in implementation have been identified and knowledge translation plans were established to engage them regularly	Easier
**Attributes of adopting organizations**	• Training material and protocols are available to guide implementation on a facility level	Easier
	• High number of births and low number of available healthcare providers	Harder
	• Providers are familiar with Robson’s Classification but do not know how to interpret the data	Neutral
**Socio-political context**	• The reduction of CS not perceived as a priority on a national level except for Thailand	Harder
	• Financial incentives for CS exist in Thailand and Viet Nam	Harder
	• Political will to implement labour companionship exists in Argentina and Burkina Faso only	Harder
	• Legal context and health workers’ fear of being sued (in Thailand and Argentina)	Harder
	• Covid-19 pandemic has a negative influence on labour companionship practices	Harder

### Attributes of the innovation

#### QUALI-DEC’s relevance and simplicity

Interviews with healthcare providers and women showed that the intervention was perceived as relevant and aligned with the needs of the target groups. Healthcare providers were familiar with audit and feedback and considered audit and feedback for CS an add-on to their existing practice. The decision analysis tool (DAT) was also perceived as an important component, especially by women who welcomed the fact that the DAT meant they could access information to make decisions.

“*Well; I think it could be useful because if the woman is informed about the advantages and disadvantages of vaginal delivery and so on the other side the upper way; she can say yes I want this or that.*” (Pregnant woman, Burkina Faso).

The benefits of labour companionship were well understood and valued among women and providers. Healthcare providers perceived the presence of a labour companion to reduce their workload especially in providing emotional support.

*“Because some of them, when they are by your side, cajole you, comfort you and that makes the woman feel good*.” (Pregnant woman, Burkina Faso).

“*I think it’s good if the family can be with women, it will help reduce our working process. Just giving a back massage should be good enough. It’s like having someone help us attend because they will sit at the bedside.”* (Labour nurse, Thailand).

However, providers expressed several challenges to implement companionship, especially with the constrained physical layout and minimal privacy in labour wards. Overall, DAT and labour companionship components were perceived as more demanding compared to audit and feedback and opinion leaders, since they required more complex changes to the organizational structure of the healthcare facilities.

## Attributes of implementers

### Reputation and credibility of implementing teams

Attributes of implementers relate to various levels of implementation. Overall, the implementing partners are well known and reputable in their disciplines. In addition, many study facilities are leading institutions and training centers. Both characteristics ease scalability of QUALI-DEC.

At a facility level, the implementation of QUALI-DEC is overseen by opinion leaders, a critical input within QUALI-DEC, and carried out by healthcare providers. Engagements with opinion leaders were context specific and depended on the level of engagement of various actors at the country level. However, as a general rule, the country teams held multiple meetings with the opinion leaders in each of the hospitals to discuss the four components of the intervention. In return, opinion leaders discussed with the healthcare providers in their hospitals to align decisions with their contexts and needs.

Selection of the right opinion leader to maximize acceptance among health providers was a challenge. Providers expressed that opinion leaders must have certain skills to ease implementation and ensure acceptability of their roles, including professional seniority and communication skills. For example, in Argentina, the assigned opinion leaders were trusted senior obstetricians and midwives, which created a credible environment for the implementation of QUALI-DEC.

*“I think, there should be the person who has the most expertise and experiences on patient care. Be the person who raises the problem and says, ‘let’s try what we should do to decrease the problem and develop a CPG’. So mostly would be the head of the department.” *(Healthcare provider, Thailand)*.*

During the development of the project, PIs requested that opinion leaders be offered financial incentives for their engagement, which we interpreted as not sustainable beyond the intervention.

### Consultations and collaborations beyond implementing teams

The stakeholder mapping and analysis at country level shows that relevant stakeholders, such as women, providers or hospital administrators were included in the design phase of QUALI-DEC intervention [15]. However, the scope of implementers extended beyond the participating facilities to other local stakeholders who were engaged in the design and implementation of QUALI-DEC. Additionally, KT plans were built leveraging ongoing partnerships with local non-governmental organizations (in Argentina), professional associations (in Thailand), international organization such as UNFPA (Viet Nam) and governmental agencies (Ministries of Health, in all countries). We interpreted these partnerships as possible facilitators in the scalability of QUALI-DEC, in terms of enhancing buy-in and ownership. Each country team had a series of online and in-person meetings throughout the intervention. The main objective of these engagements was to bring to the attention of the policymakers the growing issue of unnecessary interventions while bringing forward the perspective of the women in the four countries. For example, in Burkina Faso, the Ministry of Health requested to extend the training on Robson classification to providers in non-participating hospitals. Similarly, in Thailand, a memorandum of understanding to reduce unnecessary CS was drafted with the Royal Thai College of Obstetricians and Gynaecologists. Additionally, training workshops for implementing Robson’s classifications were conducted in many facilities but were later stopped due to COVID-19 pandemic. The teams would have preferred to organise one or more face-to-face meetings with all the stakeholders at the same time. However, this was difficult, partly because of the Covid-19 pandemic and partly because of the difficulty of bringing all the actors together.

## Attributes of adopting organizations

### Capacities of adopting facilities

Key clinical protocols were in place in most participating facilities. Available clinical protocols were either national (e.g., Argentina, Burkina Faso and Viet Nam) or adapted from national guidelines (facilities in Thailand). The presence of clinical protocols may ease implementation and integration of intervention components into already existing systems, which positively influences scalability. However, in Viet Nam the use of pre-existing clinical protocols in the hospitals created tensions as they were at odds with more recent WHO clinical algorithms creating a feeling of uncertainty within the national implementation team. The QUALI-DEC intervention did not impose any clinical guidelines on a country level.

The ultimate engagement of opinion leaders in the QUALI-DEC implementation depended on their time availability and motivation. Most study facilities reported high workload and time constraints as barriers to implementing the components of the QUALI-DEC intervention. This might make scalability of QUALI-DEC intervention harder if opinion leaders are not in place and additional human resources are needed to facilitate implementation.

Lack of space and privacy were also commonly reported as barriers towards the implementation of companionship. Across countries, implementing teams recognized that implementation of labour companionship would require a collective effort and readiness to change among providers, which could make scalability of this component harder, given the deviation from providers’ operational culture.

*“The space is tight, sometimes using the curtain is still not enough. It’s jam, many staff, no private space. If someone can come, but some may not have a companion. It may cause some inequality.” *(Labour nurse, Thailand)*.*

“*I am very afraid of the risk of confidentiality violation. The companions might talk about other patients to other people. I am very worried about this.” *(ANC nurse, Thailand).

In Burkina Faso, study facilities did not offer antenatal care for low-risk women, which was considered as a barrier in the implementation of the DAT component in the intervention. We considered this as a significant barrier towards scale-up since it may interfere with the woman’s continuity of care (discussing birth preferences with a different provider in a different facility than the one they would give birth in). Additionally, it means that the scaling-up unit would change from expanding to similar facilities to primary health care facilities which may be harder, given the different actors involved in managing these lower-level facilities compared to hospitals. From an evaluation standpoint, it would also be difficult to evaluate the effect in Burkina Faso given the changes in care provisions and settings.

## Socio-political context

### Legal and policy environment for the intervention

Findings of our review of the legal disputes (i.e., families accusing the obstetrician or clinic for misconduct) suggest that legal disputes around labour were rare and often resolved, before going to court, in Burkina Faso and Viet Nam. This was not the case for Argentina and Thailand where legal disputes followed a more structured process (see Additional file 4 on legal structures). These structures may support the use of CS especially in contexts where obstetricians are afraid of being sued in case of adverse outcomes; a factor that may influence the scalability of QUALI-DEC. However, healthcare providers in Thailand perceived “adhesion to guidelines” as a sort of insurance policy to avoid litigation.

“The lawsuit against healthcare workers is more often. So, I think, from now on we have to be careful. Whatever we do, we have to follow the guideline.” (Doctor, Thailand)

Similarly, healthcare providers in Thailand reported being afraid that labour companions may witness and report poor care towards the women- which may also be a threat to the scalability of this component, given the pronounced legal context in Thailand.

In Thailand and Viet Nam, the universal healthcare system provides financial coverage for vaginal and caesarean births, but differences in reimbursement between public and private sectors create incentives for CS. Obstetricians are reimbursed per birth performed, whereby they are reimbursed a higher amount for CS births compared to vaginal births, especially in private facilities.

*“Instead of getting a few hundred thousand Vietnam Dong in a public hospital, they receive millions from a caesarean performance at private hospitals” *(Leader of private facility, Viet Nam)*.*

In Burkina Faso, to improve access to caesarean section, user fees for caesarean section have been removed in all public hospitals since April 2016. Although the national caesarean section rate remains very low, facility-based caesarean section rates are steadily increasing with unclear medical justification.

The document review showed a mixed pattern in relation to policy support to address the overuse of CS and implement the intervention at scale. Thailand was the only country with a policy to reduce unnecessary CS. We identified national standard guidelines by The Royal Thai College of Obstetricians and Gynaecologists on improving maternal and new-born health and the quality of maternal services. We were not able to identify policies to reduce CS rates in Argentina. Instead, the Ministry of Health acknowledged that caesarean section should not be considered as a surgery on request. Similarly, in Viet Nam no legal or regulatory frameworks regarding the use of CS were available at national or subnational levels. Guidance for healthcare providers on CS on maternal request was not available in any of the four countries (Table 3). Labour companionship was not politically supported at a national level in Thailand and Viet Nam but strongly supported through laws and national policies in Argentina and Burkina Faso respectively.

**Table 3:** Available national policies and guidelines per country

National Policy/ guideline	Argentina	Burkina Faso	Thailand	Viet Nam	Influence on scalability
Reduction of CS overuse	No policies identified^a^	National priority to improve access to CS in view of low rates – but also focus on overuse of CS	A national policy to reduce unnecessary CS	No policies identified	Little support to intervention at scale where no policy is available
CS on maternal request	No policies identified	No policies identified	No policies identified	No policies identified	DAT may be perceived as a strategy to address this and there is a favourable openness for this component
Pain management for labour and CS	Pain management guidelines but use is restricted	Non-pharmacological pain management during birth and use of analgesics and anti-inflammatory drugs after CS	There is guidance but epidural is not available widely	No guidance but epidural anaesthesia is available and universally offered in three facilities	Variety of pain management initiatives present but may not be efficient and can incentivize opting for CS in some countries

*CS* Caesarean section

^a^law proposal was submitted by the implementing team in Argentina but not launched yet

The limited availability of guidelines and policies surrounding the reduction of CS, CS on maternal request and pain management could pose a challenge towards scalability of the DAT and labour companionship components of intervention in some countries (Table 3).

Country PIs expressed that the COVID-19 pandemic had a negative influence on scale-up, particularly for the labour companionship component, due to different restrictions in the labour wards (e.g. shortened visiting hours, not allowing companions to be present).

### Perceived scale-up scenarios by country principal investigators

Considering the contextual factors, implementing partners perceived scale-up differently. There was an agreement that for QUALI-DEC to be scaled up, the components of the intervention will need to be integrated in routine care and supported by national policies. Country PIs expressed interest in expanding to more facilities and within already participating facilities, for example involving more health workers in the training workshops. They also expressed opportunities to scale up two components on DAT and OLs. Two of the scale-up scenarios were (1) implementing DAT at the community level with further outreach efforts that would target more women or in primary health care facilities that offer antenatal care, and (2) recruiting more opinion leaders to expand the intervention to more healthcare professionals. There was a consensus among all implementing partners, regardless of scale-up scenarios, on the need to sustain implementation efforts in all participating facilities to have a strong demonstration of effects.

### Early adaptations in relation to scalability

Adaptations to the QUALI-DEC intervention components were introduced in each country (Table 4), with the aim to align the intervention components with the socio-political and facility-level contexts in which they are implemented, facilitate implementation and ease scalability. The main adaptations included: identification of more than one opinion leader at the facility level due to the high workloads; adoption of clinical guidelines; mobile application of the DAT; and development of IEC materials on labour companionship for pregnant women.

**Table 4:** Adaptations introduced per QUALI-DEC component in the four countries

Component	Early Adaptations				Influence on scalability
	**Argentina**	**Burkina Faso**	**Thailand**	**Viet Nam**	
**Stakeholder training**	July 2022	August 2021	December 2021	April 2022	
**Opinion Leaders** Identification of 1 opinion leader	• OLs are a senior obstetrician and a senior midwife • Tailored training material for OLs	• Having two obstetricians as OLs instead of one • Tailored training material for OLs	• Tailoring training material for OLs	• At least 4 OLs per facility; one per intervention component* • Tailoring training material for OLs	• Intervention adjusts for workload by having more than one OL • Intervention requires facilitation by OL with financial incentives to compensate for loss of incomes which questions the sustainability of practice beyond QUALI-DEC intervention
**Audit and Feedback** Implementation of audit cycles quartarely	• Adoption of a common set of clinical algorithms to standardize practice and to be used for the audit and feedback • Integration of implementation checklist to improve registration of data	• Carrying out Robson classification manually (from paper-based medical records) instead of electronically • Adoption of a common set of clinical algorithms to standardize practice and to be used for the audit and feedback	• Collecting extra variables on (birth outcome; private/non-private service) • Translating material on the WHO/QUALI-DEC platform	• Carrying out Robson classification manually in small facilities and electronically in bigger facilities • Adopting common set of clinical algorithms to standardize practice and be used for audit and feedback	• Material adaptation to make intervention more fit to context • Audits for CS are an add-on practice but require additional efforts to set-up and probably different teams to implement than those involved in the usual CS which questions simplicity of practice
**Decision Analysis Tool** Implementation of DAT	• Adapting format of DAT from paper based to digital • Material adaptation including revision of DAT booklet content, particularly wording, messages on benefits and risks per delivery mode • Adding information on pain management and role of labour companions • Development of the DAT App in Spanish	• Adapting format of DAT from paper based to digital • Integration of audios and videos in local languages and booklets with pictures for women who cannot read • Implementing DAT in primary health care facilities (although to a limited extent)	• Adapting format of DAT from paper based to digital • Tailoring DAT booklet content and wording to include messages on benefits and risks per birth mode and rating scales • Adding information on pain management and role of labour companion • Translating revised DAT materials into Myanmar language** • Developing DAT App in Thai and Myanmar language	• Adapting DAT format from paper based to digital • Integrating videos in local language • Developing DAT App in Vietnamese	• Material adaptation to make intervention more fit to context • DAT implementation in primary health care facility included changes in already existing structures
**Labour companionship** Implementation of WHO recommendations	• Develop IEC material for companionship	• Adapting WHO recommendations to the local context: Most women do not choose their companions • Installing curtains and chairs for companions in labour ward	• Adding information on labour companionship in DAT tool • Providing training on labour companionship to pregnant women and their companions via parenting school during ANC at the facility	• Developing IEC material on labour companionship for women	• Implementation requires organizational changes (values and perceptions), adaptations in place tackle these barriers and aim to make component more fit to context

^*^In some facilities with more than 20,000 deliveries per year, there is one main OL and assistants for maternity sub-units; *OL* Opinion leader

^**^around 30% of birthing women were from Myanmar; *DAT*: Decision-analysis tool

## Discussion

Our pre-implementation scalability assessment of the 4-component QUALI-DEC intervention indicated three critical factors. First, the main aim of the intervention, reducing the overuse of CS, is not a policy priority in the study countries, except for Thailand. Second, current health policies and legal frameworks do not support the intervention at scale. Instead, several incentives potentially increasing the use of CS exist, including legal (fear of litigation) and financial incentives. Thirdly, no country had guiding documents for healthcare providers on managing CS on request, despite their perceptions that practice within guidelines would protect them from litigation. Facilitating factors were that Principal Investigators and Opinion Leaders were well known in their areas of practice providing strong credibility to the QUALI-DEC intervention.

### Socio-political context and the importance of KT plans

Our analysis pointed to the dissonance between the policy and legal frameworks and the project aim. Our early assessment and realisation was used to prioritise activities within the KT plans to ensure that appropriate use of CS is raised on the political agenda [22, 23]. The QUALI-DEC intervention used an innovative approach, whereby implementation of KT plans formed a major part of the intervention approach. This included giving attention to intervention scalability from the conception of the study [3]; inclusion of state and non-state actors in the KT plans [24], and continuously targeting and engaging key actors through design and implementation [4, 25], to overcome identified bottlenecks, such as the lack of clear policy and guidance on a national level [5].

We recognise that developing a scale-up strategy requires more than strategic stakeholder engagement through KT [26, 27], particularly given the complexity of factors hindering scalability, namely financial and legal incentives supporting CS. However, given the nature of the study (a trial assessing the effectiveness and cost-effectiveness of the intervention) and resources available, this approach seems proportionate at this stage.

### Attributes of adopting organizations: Mixed ease to scale counteracted by a weak gender lens

Our analysis highlights that while women perceived the DAT and labour companionship as very relevant and useful interventions, this perception was not shared by health care providers in most settings, as they demand structural changes – as underscored elsewhere [28]. This may be a consequence of an approach lacking a robust gender lens and consideration of women’s views and needs in the planning of the provision of care. While efforts to continuously adapt implementation to better fit the context and enhance scalability are important (i.e. building on early adaptations to facilitate the introduction of these innovative elements of the intervention) [1, 6, 29], a stronger gender lens in developing scale-up strategies is warranted [30]. Within the scope of QUALI-DEC, county teams are committed to amplifying women’s voices while promoting supportive behaviours. A promising example for scale-up is in Thailand, where QUALI-DEC is prioritising the introduction of the DAT in routine antenatal care materials in the hospitals combined with intense communication targeting women and their families for decision making around CS, including through social media. This strategy enhances decision making by putting women and their families at the centre while educating and normalising choice on vaginal delivery where CS is not medically indicated.

### Attributes of the implementors: Critical yet challenging to sustain

Our analysis indicated that a local champion (named “opinion leader” in this intervention) was critical. The financial incentive for opinion leaders adopted in this project may not be sustainable if not supported by the Ministry of Health. Other means of motivating future local champions will need to be found without relying solely on monetary resources. Professional associations could be engaged through KT plans as a strategy to reinforce recognition of local champion among peers [31]. A reduction in clinical activities and protected work time for the implementation of non-clinical interventions would be a potential motivator for local champions. For this reason, facility managers could have a key role to play in scaling up the QUALI-DEC intervention.

### Strengths and limitations

Our analysis has strengths and limitations. We included a description of the iterative approach to design an intervention that is fit for scale and of the built-in processes to promote stakeholder engagement and, potentially, scalability. We used a comprehensive scalability assessment framework which included important factors for scale-up based on a rapid review of available frameworks. Multiple data sources for our analysis including a formative research and discussions with implementing partners through a training workshop session were used to populate the dimensions of the framework. Assessing scalability before the start of implementation strengthens the view on critical factors of the socio-political context and allows for an early identification of potential barriers and enablers that might affect scale-up. It also gives room to identify relevant adaptations to ease implementation and further align the intervention with local needs. The identification of critical factors strengthened the KT plan and supported the development of an adapted stakeholder engagement, which fostered trust and collaboration while allowing for the incorporation of diverse perspectives. More broadly, this proactive approach contributes to the enrichment of the field of implementation science by informing the design and implementation of more robust and nuanced health interventions while optimizing resource allocation [32].

Our study has a comparative lens, drawing on experiences of four very diverse contexts across three continents. We acknowledge that the participating facilities are not representative of the countries and further consideration would be needed on how the contextual factors, that make scale-up harder, play out in other facilities, despite the ambition to expand the intervention to more facilities. Our analysis on financial and legal incentives is limited to the health providers’ perspective relating to their clinical practice without going into nuanced hospital level clinical practices, financing modalities of private services or detailed legal stimuli [33]. Finally, we did not include primary research with stakeholders beyond implementing partners and research teams.

### Impact on research and practice within QUALI-DEC

While guidance on scale-up encourages the use of scalability assessments, this remains one of the few papers documenting implementers’ experiences in considering scalability from the start, providing a rich example of what it can mean in practice, to make an intervention “fit for context” [34, 35]. As an ex-ante scalability assessment, we do not yet have data on the effectiveness of the intervention or its cost effectiveness, two of the most critical factors to influence scale-up. These evaluations will be conducted after implementation is finalized and it would be crucial to consider the results in terms of scale-up.

Assessing the potential for scale-up also requires a deeper reflection on lessons learned from implementation, for example the extent to which adaptations designed to ease intervention implementation proved to be acceptable to target groups, and whether strategies to continue the intervention beyond the project materialised as planned e.g. making audit and feedback part of routine practice through OL facilitation. Some of the socio-political factors relating to scalability may also change during implementation, for example political champions challenging the overuse of CS.

The policy and legal discrepancies across the four countries would require additional contextual adaptations of the intervention and might lead to uneven implementation of the intervention with varied levels of adoption and support. In this case, stakeholder engagement becomes more important for aligning the intervention with the political priorities and needs of service users [36, 37]. In Burkina Faso, the issue of unnecessary CS co-exists with other very pressing public health priorities, such as the high maternal mortality due to inadequate access to life saving CS [38, 39]. This can warrant focusing a potential scale-up of the QUALI-DEC package in high volume facilities, where the benefit of audit and feedback as well as companionship may influence other birth outcomes through safe care at birth [40, 41]. This may create further support for both the policy issue (reducing unnecessary CS) and the intervention approach.

QUALI-DEC communication efforts will also aim to raise the profile of overuse of CS as a quality of care issue, while raising awareness among women on the risks associated with CS and placing responsiveness to women’s perception of quality at the heart of decision-making for CS. Therefore, an analysis of the potential for scale-up remains part of the ongoing process evaluation, through (i) in-depth interviews with Opinion Leaders at a facility level, as well as women and healthcare providers, (ii) cross-sectional survey among post-partum women, and (iii) monitoring visits in the healthcare facilities [11].

## Conclusion

Thinking about scale from the start is a key feature of the QUALI-DEC intervention. This feature has facilitated our scalability assessment due to the generation of sufficient contextual data from our formative research and supported early adaptations.

Our study provides a clear argument in favour of the use of scalability assessment frameworks as tools to systematically assess interventions, particularly to strategize activities in the KT plans and align interventions to the socio-political contexts which they are implemented in. This underscores the importance of conducting scalability assessments within a team whereby, relevant factors are discussed and interpreted with relevant actors.

## Supplementary Information

Supplementary Material 1.

Supplementary Material 2.

Supplementary Material 3.

Supplementary Material 4.

## Data Availability

The datasets used and/or analysed during the current study are available from the corresponding author on reasonable request.

## Abbreviations

WHO: World Health Organization
QUALI-DEC: QUALIty DECision-making by women and providers for appropriate use of caesarean section
CS: Caesarean Section
OL: Opinion Leader
KT: Knowledge Translation
PI: Principal Investigator
IEC: Information, Education and Communication
DAT: Decision Analysis Tool
