# Supplementary material for: Planning for scale: analysis of adaptations and contextual factors influencing scale-up of the QUALI-DEC intervention to optimize caesarean section use
Source: Implement Sci Commun. 2025 May 21;6:61. doi: 10.1186/s43058-025-00737-6 (PMC12093684; doi:10.1186/s43058-025-00737-6)
Supplement: Supplementary file 2 — Supplementary Material 2. [file 43058_2025_737_MOESM2_ESM.docx]

Table 2. Data sources for scalability assessment

| **Document review^1^** | **Readiness assessment^2^** | **Qualitative interviews^2^** | **Project reports^2^** |
| --- | --- | --- | --- |
| - Health system characteristics - Education and training of health professionals - Guidelines and protocols - Health financing - Legal context | - Inventory of physical space and readiness - Protocols and guidelines for managing clinical care during labour and childbirth - Access for audit and feedback - Labour companionship in practice | - Summaries of interviews with opinion leaders, providers and women on relevance and feasibility of implementation and legal context | - Reports of stakeholder training sessions with opinion leaders and data collectors - Knowledge translation plans on country level |

*^1^Based on secondary review of resources from the formative research; ^2^Based on data generated by the implementing partners*
