# Supplementary material for: Planning for scale: analysis of adaptations and contextual factors influencing scale-up of the QUALI-DEC intervention to optimize caesarean section use
Source: Implement Sci Commun. 2025 May 21;6:61. doi: 10.1186/s43058-025-00737-6 (PMC12093684; doi:10.1186/s43058-025-00737-6)
Supplement: Supplementary file 3 — Supplementary Material 3. [file 43058_2025_737_MOESM3_ESM.docx]

**QUALIDEC Scalability assessment checklist – v.3 October 2020**

| **Scalability dimension** | **Scalability categories** | **Detail** | | **Note** |
| --- | --- | --- | --- | --- |
|  |  |  |  |  |
| **Attributes of intervention** | **Credibility and observability of results** | 1 | The intervention is based on sound evidence | *Could not be assessed at time of analysis* |
|  |  | 2 | The effect size is likely to be of policy significance. |  |
|  |  | 3 | Causal pathway is understood and assumptions underpinning intervention chain hold true |  |
|  |  | 4 | There is evidence that the intervention works in diverse social/organisational contexts |  |
|  | **Relevance of intervention** | 5 | The intervention addresses an objectively significant, persistent health or service delivery problem |  |
|  |  | 6 | The intervention addresses an issue which is currently high on the policy agenda |  |
|  |  | 7 | The intervention addresses a need which is sharply felt by potential beneficiaries |  |
|  |  | 8 | The target group’s needs and concerns have been researched and taken into account in the intervention design |  |
|  | **Relative advantage over existing practice** | 9 | Current solutions for this issue are currently considered inadequate or are not available |  |
|  |  | 10 | If alternative solutions exist, the intervention has demonstrated superior effectiveness, acceptability, sustainability to current or alternative interventions. |  |
|  | **Simplicity** | 11 | The intervention contains few components easily added on existing practice |  |
|  |  | 12 | The components and activities of the intervention have low technical sophistication, or an easily replicated implementation pathway |  |
|  | **Transferable, testable intervention** | 13 | The intervention is being tested in the variety of service-delivery points, institutions and socio-cultural settings where it will be scaled up | *Could not be assessed at time of intervention* |
|  |  | 14 | The intervention includes little facilitation, supervision or monitoring for effective implementation, and this can be built into existing systems relatively easily and maintaining quality. |  |
|  | **Affordability**  **Cost-effectiveness** | 15 | The intervention requires a small commitment of funds for implementation at scale or generates its own financial resources for scale up | Could not be assessed at time of intervention |
|  |  | 16 | The level of financial support anticipated for scale up can realistically be mobilized |  |
|  |  | 17 | The cost-effectiveness of the intervention compared to other solutions is clearly established |  |
| **Attributes of implementers** | **Credibility of implementing team** | 18 | The proponents of the intervention are reputable organisations, with a strong technical track record and an established relationship of trust in the system where scale up is expected |  |
|  | **Local champions** | 19 | Leaders from target organisations are involved in the design and implementation of the pilot |  |
|  | **Consultation and collaboration** | 20 | In the design of the intervention pilot, input has been sought from a range of relevant stakeholders (e.g. policy-makers, managers, providers, target group) |  |
|  |  | 21 | Mechanisms to build ownership from all relevant stakeholders and institutional buy-in during implementation have been identified and set up, building on local champions networks |  |
| **Attributes of adopting organizations** | **Alignment** | 22 | No new or additional systems, infrastructure or human resources are required to implement at scale, or it is realistic to assume that these will be in place |  |
|  |  | 23 | Scale-up does not require a major changes to institutional or organizational structures or processes |  |
|  |  | 24 | The intervention is consistent with or a small departure from norms, values and operational culture of the target groups and adopting organisation(s) |  |
|  |  | 25 | The intervention presents synergies and is coherent with other interventions or programmes on similar areas involving the target group or organisations. | *Could be assessed at time of analysis* |
| **Socio-political context** | **Scope for scale-up** | 26 | The scalable unit^[[1]](#footnote-1)^ is clearly defined |  |
|  |  | 27 | The expectations for implementation “at scale” have been agreed with institutional stakeholders. |  |
|  | **Political and regulatory context** | 28 | Strong and durable political will to address problem |  |
|  |  | 29 | The policy and legal framework (including financial, economic and procedural incentives) supports implementation of the intervention at scale |  |
|  |  | 30 | If policy, regulation and legal changes are required to support implementation of the intervention at scale, it is reasonable to assume that these may be easily achieved |  |
|  | **Stakeholder engagement and sharing of learning** | 31 | A stakeholder analysis has been completed to inform the development of a scale-up strategy |  |
|  |  | 32 | There is an established mechanism for continuous engagement of external stakeholders to discuss research findings |  |
|  |  | 33 | The established mechanism has identified clear points at which the implementation process will be reviewed and adapted to improve its contextual and health system fit and promote scale up |  |

1. Defined as the smallest representative slice of the system targeted for full scale implementation. (Massoud, 2004; Barker, 2016) [↑](#footnote-ref-1)
