# Supplementary material for: Planning for scale: analysis of adaptations and contextual factors influencing scale-up of the QUALI-DEC intervention to optimize caesarean section use
Source: Implement Sci Commun. 2025 May 21;6:61. doi: 10.1186/s43058-025-00737-6 (PMC12093684; doi:10.1186/s43058-025-00737-6)
Supplement: Supplementary file 4 — Supplementary Material 4. [file 43058_2025_737_MOESM4_ESM.docx]

**Legal context in Argentina and Thailand based on secondary review of national policies**

|  | **Argentina** | **Thailand** |
| --- | --- | --- |
| **Common disputes** | - Obstetric violence - Maternal/neonatal complications and death | - Maternal/neonatal complications and death |
| **Party held accountable** | - Public hospital   Government and hospital   - Private hospital   Team leader and hospital | - Public hospital   Government and doctor   - Private hospital   Doctor and hospital |
| **Judicial party processing disputes** | --- | - Thai Medical Council - Civil Court - Criminal Court |
| **Party suing** | All wealth strata | All wealth strata |
| **Financial assistance** | - Medical malpractice insurance - Lawyers - Associations | - Lawyer council - NGOs |
